# Cinnamaldehyde Ameliorates Aluminum Oxide Nanoparticle‐Induced Cognitive Impairment by Modulating ERK and p38 Signaling in Mice

**DOI:** 10.1002/alz70855_097130

**Published:** 2025-12-23

**Authors:** Maryam Moosavi

**Affiliations:** ^1^ Nanomedicine and Nanobiology Research Center, Shiraz, Fars, Iran (Islamic Republic of)

## Abstract

**Background:**

Aluminum (Al) exposure has been implicated in Alzheimer's Disease (AD) pathogenesis. Al nanoparticles (AlNPs) exhibit enhanced neurotoxicity compared to Al ions, posing a significant concern. Cinnamaldehyde (CNMA), a natural compound with neuroprotective properties, was investigated for its ability to mitigate AlNP‐induced cognitive deficits and modulate underlying signaling pathways.

**Method:**

Adult male Swiss mice were administered AlNPs (10 mg/kg, oral) alone or in combination with CNMA (100, 200, or 300 mg/kg, intraperitoneal) for 5 days. Cognitive function was assessed using the novel object recognition (NOR) test. Western blot analysis was employed to evaluate the expression levels of phosphorylated and total ERK and p38 proteins in the hippocampus.

**Result:**

Results demonstrated that AlNP exposure significantly impaired cognitive function in mice, as evidenced by decreased discrimination index in the NOR test. Concomitantly, AlNP treatment led to an increase in phosphorylated ERK and p38 levels in the hippocampus, indicating activation of these signaling pathways. Notably, CNMA treatment, particularly at the 300 mg/kg dose, significantly ameliorated AlNP‐induced cognitive deficits and attenuated the activation of both ERK and p38 signaling pathways.

**Conclusion:**

These findings suggest that CNMA exerts neuroprotective effects against AlNP‐induced cognitive impairment, potentially through the modulation of ERK and p38 signaling pathways in the hippocampus. These results highlight the potential therapeutic implications of CNMA as a natural intervention for mitigating the neurotoxic effects of AlNPs.

